# Transient Recovery of Complete Atrioventricular Block Due to Maternal Anti-SS-A Antibody Through Antenatal Steroid Administration After 27 Weeks of Gestation

**DOI:** 10.7759/cureus.36203

**Published:** 2023-03-15

**Authors:** Kuniya Ishii, Tsuguhiro Horikoshi, Masayo Kanai, Akio Ishiguro, Yoichi Iwamoto, Hirotaka Ishido, Akihiko Kikuchi, Satoshi Masutani

**Affiliations:** 1 Department of Pediatrics, Saitama Medical Center, Saitama Medical University, Kawagoe, JPN; 2 Department of Obstetrics and Gynecology, Saitama Medical Center, Saitama Medical University, Kawagoe, JPN

**Keywords:** myocarditis, steroid, ss-a antibody, atrioventricular block, fetal

## Abstract

Maternal anti-SS-A antibodies may cause complete atrioventricular block or myocardial damage in a fetus. Effective treatment for this has not been established. Although antenatal steroids may be a treatment option for anti-SS-A antibody-related myocarditis or atrioventricular block, a complete atrioventricular block is usually considered irreversible once established. Previous reports have indicated that, in cases where antenatal steroids were effective for atrioventricular block, they were administered earlier in the pregnancy. Here we present a case where maternal steroid administration initiated from 27 weeks, which is beyond the recommended optimal treatment period, was effective in altering a complete atrioventricular block to a grade I atrioventricular block.

## Introduction

A complete atrioventricular block is a major cause of persistent fetal bradyarrhythmia with a ventricular rate of less than 100 per minute [1], and more than half of fetal complete atrioventricular blocks are commonly associated with maternal anti-SS-A antibodies [2]. The pathophysiological process is believed to be associated with the immune-mediated inflammation of the conduction system or electrophysiologic interference with heart conduction, which occurs as a result of the transplacental passage of maternal anti-SS-A antibodies [3]. Atrioventricular block appears in 2-5% of fetuses or newborns whose mothers are anti-SS-A antibody positive [2,4]. Bradycardia, myocarditis, and fetal hydrops should be managed with caution, because fetal hydrops, once it occurs, may result in fetal death. If there is a progression of fetal hydrops in utero, neonatal treatment involving pacemaker implantation or anti-heart failure therapies following early delivery will be needed.

The use of antenatal steroids is a treatment option for anti-SS-A antibody-related atrioventricular block and myocarditis [5]. Antenatal steroids, at an early stage of gestation, may reduce the degree of atrioventricular block [6]. However, once a complete atrioventricular block is established, it is usually considered to be irreversible [6,7].

We report a case of a complete atrioventricular block caused by maternal anti-SS-A antibodies in which antenatal steroids were administered from 27 weeks of gestation after the optimal treatment period had passed. It was found that the complete atrioventricular block disappeared.

## Case presentation

**Figure 1:**
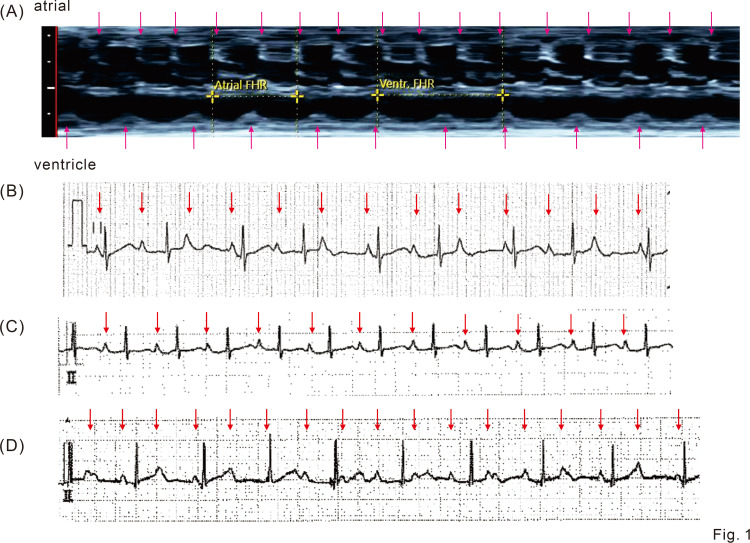
Fetal echocardiogram and postnatal electrocardiogram. (A) M-mode for an atrial and ventricular wall on fetal echocardiogram shows a complete atrioventricular block with 156 bpm of atrial rate and 101 bpm of ventricular rate. Arrows indicate atrial and ventricular contractions. (B) Electrocardiograms (lead II) just after birth. A complete atrioventricular block is displayed. Arrows indicate P wave. The paper speed is 25 mm/sec. (C) Electrocardiograms (lead II) one day after birth. The first-degree atrioventricular block is displayed. Arrows indicate P wave. The paper speed is 25 mm/sec. (D) Electrocardiograms (lead II) four months after birth. The recurrence of a complete atrioventricular block is shown. Arrows indicate P wave. The paper speed is 25 mm/sec.

**Figure 2:**
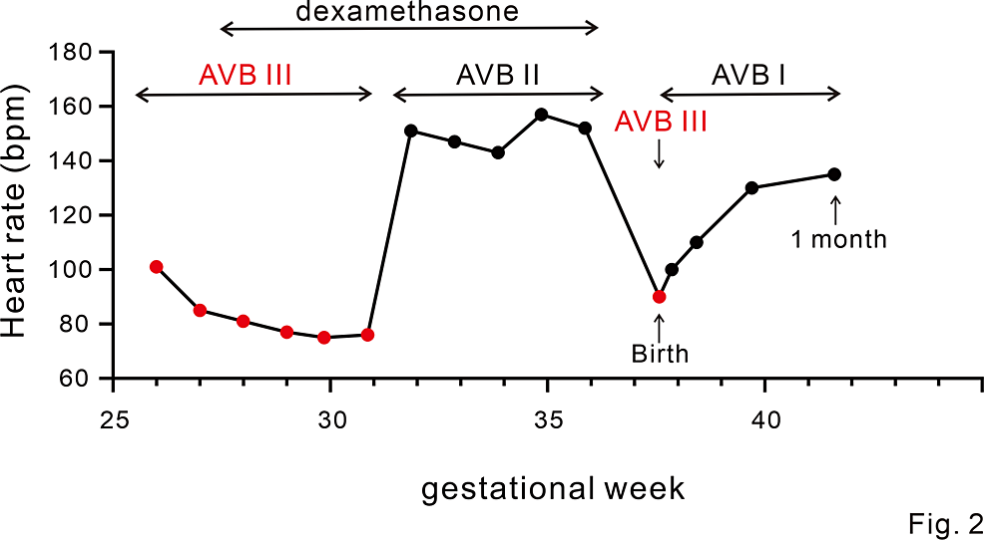
Pre- and postnatal changes of the status of atrioventricular block and ventricular rates. In parallel with dexamethasone administration, atrioventricular conduction improved to a second degree and the ventricular rate increased. Just after birth, it transiently changed to a complete atrioventricular block, but from one day of age, it changed to a first-degree atrioventricular block, and the ventricular rate was maintained. Red circles indicate a complete atrioventricular block. Black circles indicate first- or second-degree atrioventricular block. AVB: atrioventricular block.

A complete atrioventricular block, with an atrial rate of 156 bpm and ventricular rate of 101 bpm (Figure 1A and Figure 2), was diagnosed in a fetus at 26 weeks of gestation whose mother had gestational diabetes. It was subsequently revealed that the maternal anti-SS-A antibody level was high. After informed consent was obtained, oral administration of dexamethasone (4 mg/day) to the mother was initiated at 27 weeks, until 36 weeks of gestation, to prevent the progression of irreversible myocardial and conduction pathway damages. The mother was given a full explanation and she agreed to the treatment. No exacerbation of gestational diabetes was observed with the dexamethasone administration. Her QTc by Bazett correction was 0.37 s.

At 31 weeks of gestation, the atrioventricular conduction had improved to a second-degree atrioventricular block with a ventricular rate of 151 bpm (Figure 2); the second-degree atrioventricular block persisted until delivery. The baby was born by vaginal delivery at 37 weeks and four days of gestation. The birth weight was 2,265 g with APGAR scores of 8-8.

The circulatory and respiratory condition of the newborn was stable and only transient short-period support by oxygen administration and continuous nasal positive airway pressure for transient tachypnoea were required.

Just after birth, an electrocardiogram revealed a complete atrioventricular block with a maintained ventricular beat rate of 80-100 beats/min (Figure 1B and Figure 2). Echocardiography showed good cardiac contraction with a left ventricular ejection fraction of 66%. The complete atrioventricular block changed to a sinus rhythm with a first-degree atrioventricular block on day 1; a heart rate of 120 per minute was noted on day 1 (Figure 1C and Figure 2). The baby’s QTc by Fridericia correction when in first-degree heart block was 0.36 s and the baby did not have prolonged QT intervals. There were no findings suggestive of heart failure or myocarditis, and the hemodynamics were stable without requiring the use of a b-stimulant. Poor sucking was initially observed, but the mother and child were discharged from the hospital on day 15 after full oral feeding had been established and the body weight gain of the infant confirmed. We observed this infant once a week until approximately a month after discharge. Subsequently, the follow-up interval has been reduced to once a month. Four months after discharge, a complete atrioventricular block again developed. Nevertheless, the baby’s ventricular function and general condition remained stable with a ventricular rate of 90 beats per minute (Figure 1D and Figure 2). Although slightly poor weight gain was observed transiently after discharge, no developmental issue was noted on the outpatient follow-up visit. We will continue to monitor the patient carefully in the outpatient clinic.

## Discussion

In this case, the maternal steroid initiated at 27 weeks, which is beyond the optimal treatment period [7], was effective in changing the complete atrioventricular block to a grade I atrioventricular block, without the development of myocarditis or cardiac dysfunction. According to previous reports, 27 weeks of gestation is beyond the optimal timing for the initiation of an antenatal steroid [6,7]. The heart rate in this patient was maintained at 80 beats per minute or higher during the fetal and neonatal periods (Figure 2). Although there have been a few reports of maternal steroids transforming the second-degree atrioventricular block into a sinus rhythm [3], once a complete atrioventricular block is established, it is usually considered irreversible [6,7]. In a relatively large cohort (N=59), betamethasone treatment did not reverse the complete atrioventricular block [6]. In contrast, a temporal effect on atrioventricular conduction was observed in five of 29 fetuses (17%) that were first treated at ≤ 24 weeks of gestation with 8 mg/day of dexamethasone, although none of the fetuses (0%) were responsive to the treatment when it was initiated later and/or at a starting dose of 4 mg/day [7]. In all of the cases that showed an improvement of atrioventricular conduction, complete atrioventricular block recurred [2,7].

If the spread of inflammation is suppressed and the automaticity in the lower system is maintained by steroid administration, it may contribute to avoiding a single-chamber ventricular (VVI) pacemaker implantation soon after birth, even in cases where atrioventricular conduction eventually returns to the complete atrioventricular block. It is preferable to allow waiting for sufficient physical growth before implanting a dual-chamber (DDD) pacemaker [6] because the DDD pacemaker is less likely to evoke pacemaker-induced cardiomyopathy than the VVI pacemaker.

The clinical course of the current case may indicate the positive effect of an antenatal steroid in ameliorating a complete atrioventricular block, even after the optimal period for its administration has passed. However, the side effects of antenatal steroids on mothers and infants should also be taken into consideration [3,4]. This infant initially showed transient poor sucking early after birth, although the possible role of the antenatal steroid in this remains unclear. It is possible that the mother’s gestational diabetes contributed to these symptoms. A prospective international multicenter study of the detailed feto-maternal effects and side effects of maternal steroids [8] is currently underway.

## Conclusions

Some fetal cases of complete atrioventricular block may respond to maternal steroids initiated at 27 weeks of gestation after the optimal treatment period has passed. An ongoing prospective international multicenter study will clarify the detailed feto-maternal effects and side effects of antenatal steroids and may specify which patients will benefit from the treatment.
